# Effectiveness of an Extension for Community Health Outcomes case-based virtual community of practice model on patient outcomes for the management of long COVID and other fatiguing illnesses: a randomized control trial

**DOI:** 10.3389/frhs.2026.1912240

**Published:** 2026-10-28

**Authors:** Sydney P. Sharp, Tianmai M. Zhang, Douglas Taren, Alexandra Zuber, Jane C. Samaniego, Elizabeth R. Unger, Jeanne Bertolli, Jin-Mann S. Lin, Joanna J. Regan, Amy J. Armistad, John D. Scott, Bruce B. Struminger, Job G. Godino, Christian B. Ramers

**Affiliations:** 1 Laura Rodriguez Research Institute, Family Health Centers of San Diego, San Diego, CA, United States; 2 Department of Biomedical Informatics and Medical Education, University of Washington, Seattle, WA, United States; 3 Department of Pediatrics, Nutrition Section, School of Medicine, University of Colorado, Aurora, CO, United States; 4 Ata Health Strategies, LLC., Washington, DC, United States; 5 National Center for Emerging and Zoonotic Infectious Diseases, U.S. Centers for Disease Control and Prevention, Atlanta, GA, United States; 6 Project ECHO, University of New Mexico Health Sciences Center, Albuquerque, NM, United States; 7 Department of Medicine, University of Washington, Seattle, WA, United States; 8 Herbert Wertheim School of Public Health and Longevity Science, UC San Diego, La Jolla, CA, United States; 9 School of Medicine, University of California San Diego, La Jolla, CA, United States; 10 School of Public Health, San Diego State University, San Diego, CA, United States

**Keywords:** ECHO, FQHC, infection-associated chronic conditions, long COVID, patient reported outcomes

## Abstract

**Introduction:**

Long COVID is a heterogeneous chronic condition with symptoms that often overlap with other infection-associated chronic conditions (IACC). These clinical similarities highlight gaps in healthcare education and delivery, leaving many primary care clinicians unprepared to manage these conditions. To address this need, we conducted a two-arm, single-blind, cluster randomized controlled trial (Clinicaltrials.gov ID NCT05167227) in an under-resourced Federally Qualified Health Center primary care setting to evaluate the effectiveness of the Long COVID and Fatiguing Illness Recovery Program in improving patient outcomes with long COVID, myalgic encephalomyelitis/chronic fatigue syndrome, and other IACCs.

**Methods:**

From December 2021 through October 2023, 20 primary care clinicians were randomized in a 1:1 ratio. The intervention arm participated in weekly multidisciplinary Extension for Community Healthcare Outcomes (ECHO) case-based virtual community of practice sessions, monthly webinars, and quarterly short courses. The control arm participated in monthly webinars and quarterly short courses. A total of 590 patients were assigned to participating clinicians and completed surveys including Patient-Reported Outcomes Measurement Information System (PROMIS)-29 at baseline, 3-, 6-, 9-, and 12-months. Patient-reported outcomes from 298 control and 289 intervention patients completed surveys were analyzed using statistical tests and linear mixed-effects regression to examine between-arm differences and longitudinal treatment effects.

**Results:**

Both arms showed improvements over 12 months on multiple PROMIS scales including Fatigue, Physical Function, Pain Interference, Social Roles, Dyspnea, and Applied Cognition, approaching reference population norms at study end. A statistically significant difference was observed on pain interference, with the intervention arm having better outcomes than control at 12 months [mean (SD): 57 (11) vs. 60 (10), *P* = 0.009]. Longitudinal analyses revealed a significant treatment effect on pain interference [estimate = –1.95; 95% CI (–3.79, −0.10); *P* = 0.04] and a higher proportion of intervention patients achieved clinically meaningful improvements (≥5T-score points) compared with controls (43% vs. 29%; *P* = 0.01).

**Discussion:**

The ECHO case-based virtual community of practice model showed evidence that clinician-focused education may enhance primary care management of patients with complex fatiguing illnesses, supporting the model's value in under-resourced settings.

**Clinical Trial Registration**: https://clinicaltrial.gov/, identifier NCT05167227.

## 1 Introduction

Long COVID is a heterogeneous, chronic health condition characterized by a wide spectrum of symptoms, with over 200 reported to date, that range from mild to severely debilitating, with the potential to affect multiple organ systems (1–5). As of September 2024, an estimated 17.9% of U.S. adults have experienced long COVID, with 5.3% reporting persistent symptoms (6). The National Academies of Sciences, Engineering, and Medicine recognizes long COVID as an infection-associated chronic condition (IACC) with symptoms such as fatigue, post-exertional malaise, and cognitive dysfunction overlapping with other IACCs, including myalgic encephalomyelitis/chronic fatigue syndrome (ME/CFS) (7–9). These clinical parallels have positioned prior clinical experience and research with ME/CFS as a critical foundation for informing the management and care of individuals with long COVID (8, 10–12).

These overlaps also highlighted longstanding structural gaps in healthcare delivery for IACCs. As more patients sought care for long COVID, the evolving clinical landscape revealed significant gaps in clinician education, clinical infrastructure, and systems-level support. Primary care clinicians, often the first point of contact for patients with poorly defined symptoms, were responsible for diagnosing and managing this complex, multisystem condition. However, during the early phases of the COVID-19 pandemic, few primary care clinicians had received adequate education and training or had access to evolving diagnostic frameworks, leaving many unprepared to recognize long COVID or to validate and effectively support patients with persistent symptoms (13).

These deficits were especially pronounced in low-income and under-resourced settings, where clinicians faced additional barriers, such as limited referral networks and reimbursement constraints, and patients confronted significant logistical challenges (e.g., limited time, financial constraints, and transportation barriers) (14). Without sufficient clinical guidance or institutional support, many clinicians lacked the knowledge and tools to construct individualized recovery plans. These challenges exacerbated disparities in care access and quality (13).

In response to these challenges and to improve care for individuals with long COVID and other chronic fatiguing illnesses, Family Health Centers of San Diego (FHCSD), a large, urban federally qualified health center (FQHC) system, launched the Long COVID and Fatiguing Illness Recovery Program (LC&FIRP). Developed amid widespread clinical uncertainty and limited specialist access in resource-constrained settings, LC&FIRP built on lessons from ME/CFS and other IACCs. The program leveraged the Project Extension for Community Healthcare Outcomes (ECHO) collaborative case-based virtual community of practice model to support the management of complex, multisystem presentations through virtual case consultations and peer-to-peer knowledge exchange (15). In addition to each ECHO virtual community of practice session having at least two national long COVID and ME/CFS clinical subject matter experts (SME) from a national pool of 75+ experts, each session included input from an invited long COVID and ME/CFS lived-experience expert to ensure person-centeredness and enhance empathy. This structure not only enabled frontline primary care clinicians to access specialist-informed insights and direct input from those with lived experience but also facilitated the real-time development and dissemination of emerging best practices tailored to the evolving clinical landscape of long COVID. By adapting this approach to the specific challenges of IACCs, LC&FIRP aimed to build capacity for delivering patient-centered, evidence-informed best practices at the primary care level.

To evaluate the real-world impact of LC&FIRP, we examined the effectiveness of the program on patient-level outcomes, including physical function and symptom burden. We hypothesized that care delivered by clinicians participating in LC&FIRP, comprising weekly ECHO small group case-based collaborative learning sessions (hereafter referred to as “weekly ECHO sessions”), monthly interactive webinars, and quarterly short courses, would be associated with improved patient-level outcomes compared to care delivered by nonparticipating clinicians, who had access limited to monthly webinars and quarterly short courses. We also included a comparator group from the University of Washington (UW) Post-COVID Rehabilitation and Recovery Clinic and hypothesized that outcomes among patients of clinicians participating in LC&FIRP would approach the outcomes of patients receiving care in UW's highly resourced, multidisciplinary academic model. This study aimed to generate critical evidence on the effectiveness of improving patient outcomes using a case-based virtual community of practice model for managing long COVID, ME/CFS, and other IACCs within an FQHC setting. To date, there are very few randomized controlled trials (RCT) demonstrating the impact of the ECHO case-based virtual community of practice model (16–18). Similarly, to our knowledge, this is the first ECHO program evaluated for both effectiveness and implementation outcomes using randomization, patient-level data, and a comparison group. Two companion papers in this journal summarize the results on clinician outcomes and feasibility, acceptability and sustainability of this care model in managing long COVID (19, 20).

## 2 Materials and methods

### 2.1 Study design

The effectiveness of LC&FIRP was evaluated in a two-arm, single-blind, pragmatic, quality improvement, professional cluster, RCT conducted across primary care clinics at FHCSD (15). Clinicians were the unit of randomization, and a total of 20 clinicians were randomized in a 1:1 ratio to the intervention or control arm. Intervention clinicians had the opportunity to participate in weekly ECHO sessions, monthly webinars, and quarterly short courses. Control clinicians were offered access only to monthly webinars and quarterly short courses.

Weekly ECHO sessions were available only to intervention clinicians (*n* = 10) and included an SME-led didactic presentation on emerging evidence and best practices for long COVID, ME/CFS, and other IACCs, followed by an interactive question-and-answer (Q&A) session and case-based discussion. The didactic presentation and Q&A were recorded for clinicians unable to attend live; case discussions were not recorded because they included patient-specific clinical information. Case presentations fostered collaborative learning among clinicians and a national multidisciplinary panel of SMEs, most of whom were based at academic medical centers. SMEs were recruited nationwide from relevant disciplines, including but not limited to ME/CFS, pulmonology, neurology, psychiatry, cardiology, physical medicine and rehabilitation, and experts with lived experience. Initial recruitment occurred through study team referrals, with additional SMEs identified through professional networks and recent scientific literature.

At the time of study initiation, patients with long COVID, ME/CFS, or IACCs received usual care from their primary care clinician, including symptom management and referrals to specialists. Since scientific evidence was rapidly evolving, the trial incorporated an active educational control consisting of monthly webinars and quarterly short courses rather than standardized care. This design allowed evaluation of the benefit of the interactive, case-based ECHO model compared to large webinars or independent learning while ensuring all participating clinicians had access to the latest evidence and best practices.

Monthly webinars were open to all LC&FIRP clinicians (*n* = 20) and primary care clinicians nationwide. Each webinar consisted of a 30 min SME-led didactic presentation followed by an interactive Q&A session. All webinars were recorded and uploaded to LC&FIRP's ECHO homepage. Quarterly short courses required less than one hour to complete and consisted of online modules with presentation slides, videos, and knowledge-check questions following each module. Courses remained online until the next course was published. Over the course of the program, 89 ECHO sessions, 34 monthly webinars, and 9 quarterly short courses were delivered. No *a priori* attendance target was established. Intervention clinicians attended an average of 42% of weekly ECHO sessions offered and 38% of monthly webinars, while control clinicians attended an average of 47% of monthly webinars. Attendance at quarterly short courses among LC&FIRP clinicians was low, although the courses reached a broad national audience. Lower attendance across each educational component was due to existing clinical demands. Further details regarding clinician outcomes can be found in our companion paper (19).

Eligible LC&FIRP participants were primary care physicians, physician assistants, and nurse practitioners at FHCSD who provided care for patients with long COVID, ME/CFS, and/or IACC, and who consented to participate in the study for up to 37 months. Each clinician was assigned approximately 30 patients, resulting in a total cohort of 590 patients diagnosed with long COVID, ME/CFS, and/or IACC. FHCSD is one of the nation's ten largest federally qualified health center (FQHC) systems, serving a predominantly low-income, racially and ethnically diverse patient population (15). Outcomes of LC&FIRP patients were also compared to those from a patient cohort enrolled in the UW's Post-COVID Rehabilitation and Recovery Clinic (21). Further details regarding the study design and setting are described in the published protocol paper (15).

The study was registered on December 22, 2021 at Clinicaltrials.gov (NCT05167227) and was reviewed and approved by the Institutional Review Board of San Diego State University, approval number HS-2021-0241. This study was funded by the US Centers for Disease Control and Prevention (CDC) and was conducted by a consortium of practice-based and academic institutions that include FHCSD, Ata Health Strategies, ECHO Institute at the University of New Mexico Health Sciences Center, University of Colorado School of Medicine, and UW School of Medicine.

This study was reported in accordance with the Consolidated Standards of Report Trials (CONSORT) guidelines (22). The CONSORT checklist of this study is available in the appendix.

### 2.2 Patients

Patients were recruited through primary care clinicians at FHCSD, who introduced the study to eligible individuals and submitted referrals for those expressing interest. Recruitment occurred from December 2021 through October 2023, enrolling 590 patients. Inclusion criteria were: (1) age 18 years or older; (2) a decline in health-related quality of life and persistent symptoms related to long COVID, ME/CFS, or IACC determined by the referring clinician; and (3) completion of FHCSD's Broad Consent, including authorization for use of Protected Health Information for research purposes. Patients met one of the following diagnostic categories: (1) existing or new ICD-10 diagnosis of post-COVID condition with laboratory-confirmed SARS-CoV-2 infection, (2) existing or new ICD-10 diagnosis of post-COVID condition without laboratory confirmation of SARS-CoV-2 infection, (3) existing or new ICD-10 diagnosis of ME/CFS, and (4) diagnosis of another IACC such as fibromyalgia or postural orthostatic tachycardia syndrome. Clinicians were instructed to apply the prevailing long COVID definition at the time: “persistent symptoms four or more weeks following SARS-CoV-2 infection ” (4, 23). After a referral was submitted, a trained research assistant contacted the patient by phone to confirm eligibility by verifying that they were experiencing long COVID symptoms affecting their quality of life and to provide program information. Patients then decided whether to participate. Enrollment was voluntary, and participants could withdraw from the study at any time. Upon consent, a baseline survey was administered via phone by a research assistant. Since the intervention targeted clinicians rather than patients, patients were not randomized and were instead assigned to a participating LC&FIRP clinician. The program manager, unblinded to study arm, conducted assignments based on clinic location, patient language preference, and clinician caseload to ensure that participating LC&FIRP clinicians received equal patient loads. Research assistants remained blinded throughout the study.

Following assignment, patients were scheduled for a baseline clinical appointment, completed via telehealth or in person, based on patient preference. Calls and surveys were primarily conducted in English and Spanish, with interpreter services used as needed. Patients were followed for a minimum of 12 months, completing surveys at 3-, 6-, 9-, and 12-months. Baseline survey domains included COVID-19 experience, symptom profiles, medical history, health history, and patient-reported outcomes (PROs). Follow-up survey domains included COVID-19 experience, reassessing symptoms that were endorsed at baseline, and PROs. However, patients could report additional symptoms at any follow-up survey, recognizing the fluctuating nature of long COVID. PRO measures included the Patient-Reported Outcomes Measurement Information System (PROMIS)-29 v2.1, PROMIS Short Form v1.0 Dyspnea Severity 10a, PROMIS Applied Cognitive—General Concerns 8a, Patient Health Questionnaire-2 (PHQ-2), Generalized Anxiety Disorder-7 (GAD-7), and the CDC ME/CFS Symptom Screener—Short Form v1.2 (24–29).

Patients were scheduled for an initial appointment with their assigned LC&FIRP clinician, though completion rates varied. Treatment plans were determined at the clinician's discretion, with no restrictions on routine clinical care, and no standardized follow-up schedule. Physical therapy referrals to therapists trained in the evaluation and care of fatiguing illnesses were also offered. Harms were dealt with according to FHCSD's policies. Throughout the study, patients remained blinded to their clinician's intervention arm. A detailed patient flow diagram is provided in Figure 1.

**Figure 1:**
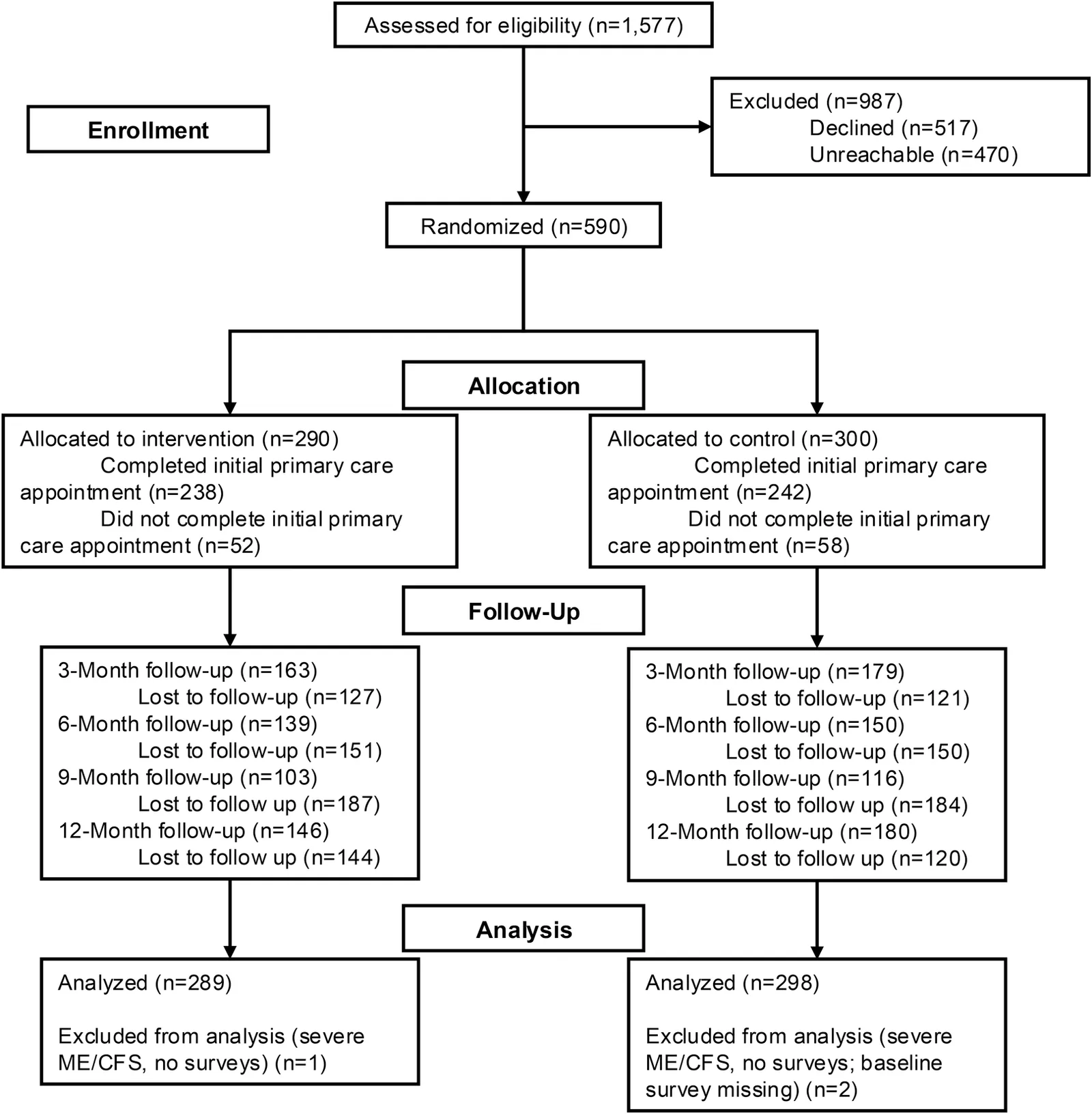
Patient consort diagram.

The external comparison cohort included patients from UW Post-COVID Rehabilitation and Recovery Clinic, which serves the five-state region of Washington, Wyoming, Alaska, Montana, and Idaho (30). The clinic used a hybrid model of in-person and telemedicine visits with seven clinicians specializing in long COVID care. Patients completed an intake survey at their first visit and a follow-up survey 12 months later. The LC&FIRP baseline survey was based on UW's intake survey, and the LC&FIRP 12-month survey informed UW's follow-up survey, ensuring aligned domains. A total of 238 UW patients completed both surveys and were included in analysis.

### 2.3 Sample size

The primary patient outcome was PROMIS-29 physical function after 12 months, sample size calculations were re-calculated based on a two-sided, two-sample *t*-test at a significance level of 5%. The literature on minimally important differences in the PROMIS-29 domains suggests it is equal to 0.45–0.5 SD (4.5–5.0 points on a T-score metric). A 0.45–0.5 SD between group difference equates to a standardized effect size d between 0.45 and 0.5. As no similar cluster randomized controlled trial has been reported in the literature and the sequalae of the illnesses of interest appear heterogeneous, we have assumed that the intraclass correlation will be low (ICC = 0.1). Adjusting for this clustering of our primary outcome between 20 clinicians, we estimate that follow-up of 280 to 300 patients (140 to 150 per condition, 7 to 7.5 per clinician cluster) will provide us with between 79% and 80% power.

### 2.4 Outcomes

The primary patient outcome was the change in physical function, measured by the Physical Function subscale of the PROMIS-29 Profile. Secondary outcomes included PHQ-2, GAD-7, PROMIS dyspnea severity, PROMIS applied cognition, and PROMIS-29 subscales of depression, anxiety, fatigue, pain interference, social roles, sleep disturbance, physical summary, and mental summary. Exploratory outcomes included the number of referrals received and number of completed encounters during patients' one-year enrollment in LC&FIRP, as determined by chart review. Outcomes scores were calculated according to standardized scoring protocols. PROMIS T-scores (i.e., standardized scores with a mean of 50 and a standard deviation of 10 in a reference population) were calculated using the HealthMeasures Scoring Service (31). PROMIS-29 physical and mental health summary scores were derived from relevant subscales, following the algorithm developed by Hays et al. (32).

### 2.5 Statistical analysis

All intervention and control arm FHCSD patients were included in the treatment effect analysis according to the intention-to-treat principle. Missing data were handled using an available case analysis approach, and no imputation was performed. Statistical differences in patient characteristics and PROs between arms were examined using the chi-squared test, Student *t*-test, or Wilcoxon test, according to data type and normality.

Linear mixed effects (LME) regression (33) was used to model longitudinal patient outcomes and estimate the average treatment effect using repeated measures from all available time points. The following R formula was used for the base LME model of each PROMIS outcome:

Score∼Time+Arm+Time:Arm+ScoreBaseline+(1|PatientID)

where Time is categorical or continuous for baseline, 3-, 6-, 9-, and 12-months, Arm is binary for intervention and control, Time:Arm is the interaction between time and arm for the estimation of average treatment effect, ScoreBaseline is the baseline outcome for addressing potential regression to the mean, and (1 | PatientID) represents an individual intercept for each patient to account for random effects. LME models with different sets of additional covariates (e.g., demographic variables) or different random effect settings were evaluated with the Akaike's Information Criteria to find the best model. Model validity was diagnosed using Shapiro–Wilk test of residual normality and White's Lagrange multiplier test of residual variance homogeneity.

Minimal important change (MIC) values of PROMIS scales were utilized to assess whether between-group differences or estimated treatment effects are clinically meaningful (34, 35). Specifically, we used the maximum MIC values estimated by previous studies or 3T-score points (the generally recommended value for between-group comparison), whichever smaller, for PROMIS scales whose maximum MIC estimates are smaller or near 3, and 5T-score points (a half standard deviation of PROMIS T-scores) for scales with larger MIC estimates. For within-group changes over time, we used larger thresholds than between-group MIC values, as recommended by the PROMIS instructions. We further analyzed the number of patients whose score changes over 12 months reached MIC thresholds in both arms.

Exploratory analysis of the moderation of intervention effects by the number of clinical encounters considered both the number of referrals received by a patient and the number of encounters the patient completed at FHCSD. Detailed methods of this exploratory, 3-way interaction analysis can be found in supplementary materials.

The cohort comparison analysis between FHCSD and UW included a subset of patients who completed both baseline and 12-month questionnaires. Patient outcomes at baseline and 12 months were compared using corresponding statistical tests, with or without weights from propensity score matching on age, sex, and COVID-19 hospitalization history to account for cohort differences in patient characteristics. In propensity score matching, the matching algorithm achieving the best matching performance was selected for weight calculation. Effect sizes, specifically Hedge's *g* for continuous variables and Cramer's *V* for categorical variables, are reported along with *P*-values to measure the magnitude of cohort differences in consideration of the large sample size. LME regression with or without weights from propensity score matching was then conducted in a similar manner as described above to estimate cohort differences in PRO changes over 12 months.

All statistical analyses were performed using R (version 4.3.3). Propensity score matching and LME regression were conducted using R packages MatchIt (version 4.5.5) and lmerTest (version 3.1–3), respectively. A *P*-value of less than.05 was considered statistically significant.

## 3 Results

A total of 590 patients were enrolled in the study. Of these, 587 completed the baseline survey: two patients with severe ME/CFS were unable to complete the phone-based survey, and one survey was lost due to a technical error. Among those who completed baseline, 298 were assigned to the control arm and 289 to the intervention arm. Patients were predominantly female (69.8%), White (69.5%), low-income (94.4% below $50,000 annually), and a large proportion were more comfortable receiving care in Spanish (40.2%), as shown in Table 1. Although we only randomized clinicians into two arms, patient characteristics were similar across study arms, suggesting effective randomization. No harms were reported to the Institutional Review Board during the study.

**Table 1:** Family Health Centers of San Diego patient characteristics^a^.

	All *N* = 587	Control *n* = 298	Intervention *n* = 289
Age, mean (SD)	47.7 (12.7)	48.4 (12.6)	47.1 (12.9)
Sex, *n* (%)			
Female	410 (69.8%)	212 (71.1%)	198 (68.5%)
Male	177 (30.2%)	86 (28.9%)	91 (31.5%)
Race, *n* (%)			
White	408 (69.5%)	213 (71.5%)	195 (67.5%)
Non-white	78 (13.3%)	39 (13.1%)	39 (13.5%)
Marital status, *n* (%)			
Married	288 (49.1%)	150 (50.3%)	138 (47.8%)
Single	195 (33.2%)	100 (33.6%)	95 (32.9%)
Separated, divorced, or widowed	89 (15.2%)	38 (12.8%)	51 (17.6%)
Education, *n* (%)			
Below grade 9	70 (12.0%)	38 (12.8%)	32 (11.1%)
Grade 9–12	194 (33.0%)	107 (35.9%)	87 (30.1%)
Some college and above	293 (49.9%)	134 (45.0%)	159 (55.0%)
Employment status, *n* (%)			
Employed	233 (39.7%)	120 (40.3%)	113 (39.1%)
Unemployed	222 (37.8%)	109 (36.6%)	113 (39.1%)
Student or retired	56 (9.5%)	24 (8.1%)	32 (11.1%)
Housing status, *n* (%)			
Own or rent	500 (85.2%)	254 (85.2%)	246 (86.1%)
Homeless	59 (10.1%)	29 (9.7%)	30 (10.4%)
Income, annual estimate, *n* (%)			
$0	171 (29.1%)	84 (28.2%)	87 (30.1%)
$1–19,999	231 (39.4%)	124 (41.6%)	107 (37.0%)
$20,000–49,999	152 (25.9%)	76 (25.5%)	76 (26.3%)
$50,000 and above	22 (3.7%)	9 (3.0%)	13 (4.5%)
Preferred language, *n* (%)			
English	342 (58.3%)	165 (55.4%)	177 (61.2%)
Spanish	236 (40.2%)	130 (43.6%)	106 (36.7%)
Diagnosis category, *n* (%)			
Long COVID only	536 (91.3%)	274 (91.9%)	262 (90.7%)
Long COVID and ME/CFS	36 (6.1%)	19 (6.4%)	17 (5.9%)
ME/CFS only	13 (2.2%)	4 (1.3%)	9 (3.1%)
Other IACCs	2 (0.3%)	1 (0.3%)	1 (0.3%)
Hospitalized due to COVID-19, *n* (%)			
Yes	139 (23.7%)	69 (23.2%)	70 (24.3%)
No	443 (75.5%)	226 (75.8%)	217 (75.3%)
Admitted to ICU due to COVID-19, *n* (%)			
Yes	78 (13.3%)	39 (13.1%)	39 (13.5%)
No	504 (85.9%)	256 (85.9%)	248 (86.1%)
Intubated due to COVID-19, *n* (%)			
Yes	39 (6.6%)	16 (5.4%)	23 (8.0%)
No	543 (92.5%)	279 (93.6%)	264 (91.7%)

a The frequency and percentage of missing values are not shown, therefore the total percentage for the indicated categories may not sum up to 100%.

Table 2 provides summary statistics of PROs of FHCSD patients every three months from baseline to 12 months. At baseline, patients in the two arms did not have statistically significant differences on any of the PROs. When comparing the two arms at each of the time points, the PROMIS-29 Pain Interference scale at 12 months was the only outcome with a statistically significant difference [mean (SD): control 60 (10) vs. intervention 57 (11), *P* = 0.009]. Both arms showed improvements in mean scores from baseline to 12 months on multiple metrics including Fatigue, Physical Function, Pain Interference, Social Roles, Dyspnea, and Applied Cognition (Table 2). Since PROMIS T-scores were calibrated such that a score of 50 indicates the mean score of the reference population (e.g., the 2000 US Census population for PROMIS-29), mean scores of both arms increased to near the reference mean at 12 months on Depression, Social Roles, and Dyspnea scales.

**Table 2:** Patient-reported outcomes of Family Health Centers of San Diego participants at each time point^a^.

	Control					Intervention				
	Baseline *n* = 298	3M *n* = 179	6M *n* = 150	9M *n* = 116	12M *n* = 180	Baseline *n* = 289	3M *n* = 163	6M *n* = 139	9M *n* = 103	12M *n* = 146
PHQ-2^b^, [0–6], > = 3^d^, *n* (%)	87 (29%)	55 (31%)	31 (21%)	21 (18%)	28 (16%)	97 (34%)	48 (29%)	26 (19%)	27 (26%)	23 (16%)
PROMIS-29 Depression^b^, [20–80]	54 (11)	54 (12)	52 (10)	51 (10)	52 (11)	54 (10)	53 (10)	52 (10)	51 (10)	52 (11)
GAD-7^b^, [0–21]	7.1 (5.8)	7.5 (6.2)	5.6 (5.2)	5.7 (5.5)	5.7 (5.3)	7.2 (5.6)	6.6 (5.6)	6.5 (5.8)	5.7 (4.8)	5.8 (5.8)
PROMIS-29 Anxiety^b^, [20–80]	57 (12)	56 (12)	54 (11)	52 (11)	55 (12)	57 (11)	56 (12)	54 (11)	53 (10)	54 (12)
PROMIS-29 Fatigue^b^, [20–80]	61 (10)	60 (11)	59 (10)	57 (13)	57 (12)	62 (10)	60 (12)	58 (12)	58 (11)	57 (12)
PROMIS-29 Physical Function^c^, [20–80]	38 (7)	37 (8)	38 (10)	39 (9)	40 (10)	37 (8)	38 (10)	38 (9)	40 (10)	41 (10)
PROMIS-29 Pain Interference^b^, [20–80]	61 (10)	61 (10)	59 (10)	59 (11)	60 (10)	61 (10)	60 (10)	59 (10)	59 (11)	57 (11)
PROMIS-29 Social Roles^c^, [20–80]	45 (11)	47 (12)	50 (12)	50 (13)	51 (12)	45 (11)	46 (12)	50 (12)	50 (11)	51 (12)
PROMIS-29 Sleep Disturbance^b^, [20–80]	57 (8)	57 (9)	56 (8)	56 (9)	57 (8)	58 (8)	57 (9)	58 (9)	55 (9)	56 (8)
PROMIS-29 Physical Summary^c^, [20–80]	37 (9)	37 (9)	39 (10)	39 (10)	40 (10)	37 (8)	38 (10)	39 (10)	40 (11)	42 (10)
PROMIS-29 Mental Summary^c^, [20–80]	41 (9)	41 (10)	44 (9)	45 (11)	44 (11)	40 (9)	42 (10)	44 (10)	44 (10)	45 (11)
PROMIS Dyspnea Severity^b^, [20–80]	57 (12)	56 (11)	53 (12)	53 (12)	51 (12)	56 (11)	54 (11)	53 (12)	52 (12)	49 (12)
PROMIS Applied Cognition^c^, [20–80]	43 (12)	44 (12)	48 (11)	47 (12)	49 (12)	43 (12)	45 (13)	45 (12)	45 (11)	47 (13)

a All data are mean (SD) except PHQ-2. Score ranges are indicated in square brackets. PROMIS T-scores have a mean of 50 and standard deviation of 10 in the corresponding reference population and typically fall between 20 and 80.

b On PHQ-2, GAD-7, and all PROMIS subscales except Physical Function, Social Roles, Applied Cognition, and summary scales, a higher score indicates more severe problems or concerns.

c On PROMIS Physical Function, Social Roles, Applied Cognition, and summary scales, a lower score indicates more severe problems or concerns.

d A PHQ-2 score of 3 or greater indicates that major depressive disorder is likely.

Besides further validating aforementioned Table 2 findings of the improvements in mean outcomes (based on LME estimates of time effects, data not shown), adjusted longitudinal LME models (Table 3) indicate a statistically significant treatment effect over 12 months on the Pain Interference scale [Time:Arm interaction term estimate = −1.95, 95% CI (−3.79, −0.10), *P* = 0.04]. However, the magnitude of this estimate does not reach the corresponding MIC value of 5, indicating that this average treatment effect might not be clinically meaningful. Addition of patient characteristic variables (e.g., age, sex, intubation history) had minimal impact on treatment effect estimates and their *P* values.

**Table 3:** Average treatment effect estimates on PROMIS scales over 12 months from baseline-adjusted linear mixed effects models^a^.

	Estimate	95% CI	*P*
PROMIS-29 Depression^b^	−0.64	[−2.46, 1.18]	.49
PROMIS-29 Anxiety^b^	−1.89	[−3.92, 0.15]	.07
PROMIS-29 Fatigue^b^	−1.16	[−3.20, 0.88]	.27
PROMIS-29 Physical Function^c^	1.49	[−0.04, 3.02]	.06
PROMIS-29 Pain Interference^b^	−1.95	[−3.79, −0.10]	.04
PROMIS-29 Social Roles^c^	−0.57	[−2.60, 1.47]	.58
PROMIS-29 Sleep Disturbance^b^	−1.60	[−3.22, 0.02]	.05
PROMIS Dyspnea Severity^b^	−1.34	[−3.69, 1.00]	.26
PROMIS Applied Cognition^c^	−1.74	[−3.68, 0.19]	.08

a Average treatment effect estimates come from the interaction term between time and intervention. These estimates reflect average relative changes in the outcomes of the intervention arm compared to the control arm.

b On all PROMIS subscales except Physical Function, Social Roles, and Applied Cognition, a higher score indicates more severe problems or concerns. Therefore, a negative treatment effect estimate indicates a positive treatment effect.

c On PROMIS Physical Function, Social Roles, and Applied Cognition scales, a lower score indicates more severe problems or concerns. Therefore, a positive treatment effect estimate indicates a positive treatment effect.

A further analysis of change scores of individual patients revealed that a significantly higher proportion of intervention patients achieved minimally important improvements on the Pain Interference scale from baseline to 12 months. Among those who completed both baseline and 12-month questionnaires (*n* = 174 in control and *n* = 145 in intervention), 43% (*n* = 63) of patients in the intervention arm reported a score improvement of at least 5T-score points on the Pain Interference scale, while in the control arm the proportion was 29% (*n* = 50), with a *P*-value of.01 from the Chi-squared test. This pattern persists when the threshold is increased to 10, one SD of PROMIS T-scores, and even 12.4, the maximum MIC estimate for the Pain Interference scale from previous studies, suggesting that more patients in the intervention arm than control experienced meaningful improvements in terms of pain interference with life (34, 35). On other PROMIS scales, no statistically significant differences between individual meaningful change proportions of the two arms were observed. Complete results can be found in Supplementary Table S1.

Exploratory analyses of the number of referrals or encounters showed that participants in the intervention arm received fewer referrals (median = 9, Q1 = 3.25, Q3 = 15 vs. median = 10, Q1 = 6, Q3 = 16, *P* = 0.008) and completed fewer encounters (median = 16, Q1 = 8, Q3 = 28.8 vs. median = 21, Q1 = 11, Q3 = 33.2, *P* = 0.002) than control participants. Both 2-way subgroup comparison and LME models with binary categories of referral or encounter frequency indicate that intervention patients who had fewer-than-median number of received referrals or completed encounters reported not only lower (better) scores at baseline but also the largest score decrease (improvement) over 12 months on the PROMIS-29 Pain Interference scale compared to patients in other subgroups. For example, intervention patients with fewer than median number of received referrals reported an average T-score of 59 (SD = 10) at baseline which decreased to 54 (SD = 11) at 12 months, whereas intervention patients who received more referrals than median reported an average T-score of 63 (SD = 9) at baseline and 60 (SD = 10) at 12 months. Complete results can be found in Supplementary Tables S2, S3. Clinician attendance at ECHO sessions or webinars was not statistically significant in arm-specific LME models of patient outcomes.

Symptom prevalence and severity results from the CDC ME/CFS Symptom Screener are available in Supplementary Tables S4, S5. At baseline, symptom prevalence ranged from 67.9% for joint pain to 94.9% for fatigue, tiredness, or exhaustion, and the two arms showed similar symptom prevalence and severity profiles. After 12 months, the severity scores of muscle pain, muscle cramps, or muscle aches reported by intervention patients become significantly lower than the control arm [mean (SD): control 7.8 (5.5) vs. intervention 5.8 (4.9), *P* = 0.01]. The mean severity score of joint pain is also lower in the intervention arm [control 7.6 (5.4) vs. intervention 6.1 (4.8), *P* = 0.06] with a near-significant difference. These results independently support the main finding from PROMIS scores that the intervention arm had greater improvements on pain-related outcomes.

### 3.1 Cohort comparison

The cohort comparison analysis between FHCSD and UW included 319 FHCSD patients (174 in control and 145 in intervention) and 238 UW patients who completed both baseline and 12-month questionnaires. Supplementary Table S6 shows that patients at the two sites have statistically similar age and sex profiles, but a significantly higher percentage of FHCSD patients had been hospitalized (22.9% vs. 8.8%, *P* < 0.001) and admitted to the ICU (12.2% vs. 2.9%, *P* < 0.001) due to COVID-19. Note that patients in the UW cohort were recruited from the long COVID clinic only, whereas recruitment for the FHCSD cohort was more broadly inclusive of IACCs including ME/CFS (Table 1).

An initial analysis of PROs compared the FHCSD control arm, the FHCSD intervention arm, and the UW cohort. Pairwise results (Supplementary Table S7) showed much larger statistical differences between the two sites than in arm-specific comparisons at both baseline and 12 months. In consideration of large cohort differences, subsequent analyses combined intervention and control at FHCSD and focused on comparisons of the two cohorts.

Propensity score matching was then conducted to resolve the observed imbalance in patient characteristics of the two cohorts. Full matching with a logit link was selected as it achieved the best matching performance (a standardized mean differences in propensity scores of 0.0012 vs. 0.5107 before matching) among all matching methods. Subsequent statistical analyses were all weighted using the matching weights from propensity score matching.

Statistical tests and terms corresponding to baseline differences in weighted LME models indicate similar differences in PROs between the two cohorts at baseline. Baseline PRO differences (Supplementary Table S8) were statistically significant on most scales due to large sample sizes, while medium effects sizes and differences above MIC thresholds were observed only on three PROMIS subscales: Fatigue, Social Roles, and Applied Cognition, indicating that UW patients reported more fatigue, less ability to participate in social roles, and lower cognitive function than FHCSD patients at baseline. After 12 months, patients in both cohorts reported improved outcomes to some extent, but the statistically significant differences on these three scales remained at 12-month follow-up (Supplementary Table S8). Regarding cohort differences in PRO changes over time (Supplementary Table S9) estimated by weighted LME regression, time-cohort interactions on Fatigue and Cognition scales were not statistically significant, indicating similar improvements in both cohorts in terms of fatigue and cognitive function. However, the two cohorts had different degrees of improvement over time on the Social Roles and Dyspnea scales, with the FHCSD cohort experiencing statistically larger improvements than the UW cohort in terms of dyspnea and the ability to participate in social roles/activities. Unweighted LME models with patient characteristics adjusted yielded similar estimates and significance levels (data not shown) as weighted LME models, indicating that the same conclusions can be reached without using propensity score matching.

## 4 Discussion

This study evaluated the effectiveness of a novel, primary care–based program designed to support the management of long COVID, ME/CFS, and other IACCs in a resource-constrained large urban FQHC clinical practice setting. Using longitudinal data collected from PROs over a 12-month period, we assessed changes in physical function and symptom burden among patients receiving care from LC&FIRP-participating clinicians. LME regression found that patients in both study arms demonstrated improvements across multiple domains over the 12-month period. Between arm-differences revealed that the intervention arm reported meaningful improvement in pain interference and required fewer referrals and overall encounters than the control arm, suggesting potential health benefits from the intervention. These results suggest that a multidisciplinary, team-based care model, centered on peer-to-peer sharing of best practices and regular virtual community of practice support from remote experts, can meaningfully enhance care outcomes in an under-resourced primary care setting. These findings highlight the promise of implementing a virtual, multidisciplinary, team-based care model within a large FQHC network—addressing a critical gap in the delivery of care for IACCs such as long COVID.

Notably, in the FHCSD cohort pain interference was the only measure to show a statistically significant difference between intervention and control arms at 12 months, with the intervention arm (43%) reporting lower (i.e., more favorable) scores compared to the control arm (29%). Although both arms improved over time, the intervention arm demonstrated a more consistent trajectory, whereas the control arm exhibited great fluctuation. This pattern may reflect the stabilizing influence of clinician participation in structured, multidisciplinary education and ongoing peer consultation. However, the between-arm difference did not exceed the corresponding MIC threshold, suggesting that while statistically significant, the average effect may not reach clinical relevance at the group level. These findings are consistent with prior research documenting heterogeneous symptom trajectories in patients with long COVID, including studies identifying distinct recovery phenotypes over six months (36). They also align with research identifying pain interference as one of the most affected PROMIS domains in this population (37).

Healthcare utilization measures such as the number of referrals and completed clinical encounters had statistically significant interactions with the intervention effect for pain interference outcomes. Compared to controls, intervention patients had fewer referrals and clinical encounters, while also demonstrating greater improvement over time. This pattern may reflect increased clinician confidence in managing complex cases within primary care, potentially stemming from the LC&FIRP's emphasis on individualized care planning and self-management support. Participation in case consultations and engagement with lived-experience experts may have further enhanced clinicians' ability to deliver targeted, empowering care without unnecessary escalation.

These findings suggest that multidisciplinary education and collaborative learning can positively influence patient outcomes, particularly in resource-constrained settings. Although previous studies have consistently reported increased healthcare utilization among patients with Long COVID, they compared individuals with Long COVID to non-Long COVID controls (38–42). In contrast, both study arms in our trial included patients with Long COVID, making the lower referral rates and fewer clinical encounters observed in the intervention arm particularly noteworthy. These findings suggest that the ECHO model may reduce healthcare utilization and associated costs by increasing primary care clinicians' capacity to manage Long COVID. This interpretation is consistent with prior work emphasizing that educating and empowering primary care clinicians to manage Long COVID can improve access to high-quality care beyond specialty Long COVID clinics (41).

Finally, to contextualize the patient population and program performance, outcomes of the intervention and control arms were compared with those from an external academic medical center cohort. Comparing this FQHC population, which is often underrepresented in Long COVID research, with an academic medical center cohort enhances the generalizability of our findings across different geographic regions, healthcare settings, and models of care. Site-level comparisons between FHCSD and the UW revealed greater variation than that observed between randomized study arms. Despite higher rates of hospitalization and ICU admission during acute infection, FHCSD patients reported better baseline and 12-month scores in fatigue, social roles, and cognitive function. Possible contributing factors include differences in care delivery models, cohort composition, referral pattern, access barriers, symptom reporting behaviors, and clinician exposure to targeted education through LC&FIRP. These findings highlight the complex and multifactorial nature of long COVID, in which the severity of acute illness does not reliably predict long-term symptom burden or functional outcomes (31–33).

### 4.1 Limitations and strengths

This study had several limitations. First, recall bias may have affected survey responses, as assessments were administered at fixed three-month intervals and conducted by phone. Patients may have had difficulty recalling experiences or may have interpreted questions differently than if the surveys were self-administered.

Second, survey length may have contributed to respondent fatigue. Baseline surveys averaged 45 min, and follow-ups 25 min. Since many participants experienced symptoms such as fatigue and brain fog, some may have become inattentive or disengaged toward the end of the survey, potentially leading to rushed or inaccurate responses. Survey burden may also have reduced retention, with 12-month survey completion rates of 50.5% in the intervention arm and 60.4% in the control arm. Consequently, we may have disproportionately lost data from patients with more severe symptoms who found the surveys too burdensome, as well as from patients whose symptoms improved and no longer felt the need to participate potentially impacting data interpretation. Future studies could explore alternative data collection methods, such as ecological momentary assessment, to allow for shorter, real-time self-reported assessments, potentially reducing recall bias and improving retention.

Third, the fluctuating nature of symptoms and their overlap with preexisting or chronic conditions make it difficult to interpret the treatment effect with certainty. Differentiating intervention impact from the natural course of illness or co-occurring conditions remains a challenge in studies involving complex, multi-system conditions like long COVID. Our study did not adjust for comorbid conditions.

Fourth, the use of an active control rather than usual care may have introduced a Hawthorne effect. Clinicians may have modified their practice because they were aware they were participating in a study and receiving study-related education. These educational opportunities likely increased awareness of long COVID, ME/CFS, and IACCs across both study arms, potentially improving patient care and diminishing between-group differences.

Fifth, since patients were assigned to clinicians based on clinic location and language preference rather than randomization, there was potential for selection bias through the clustering of pre-existing patient characteristics. To minimize this risk, the program manager regularly monitored clinician caseloads to ensure patients were distributed evenly across participating clinicians. If a patient's primary clinic was unavailable, they were assigned to the closest participating clinic. Additionally, if a clinician temporarily paused referrals or withdrew from the program, their patients were reassigned to another clinician within the same study arm. This closely mimics what occurs in routine primary care. Data analysis confirmed that patient characteristics were similar across study arms, suggesting effective assignment.

Sixth, clinician participation was limited by existing clinical demands. Many clinicians in the LC&FIRP had heavy caseloads and were unable to regularly attend ECHO sessions or webinars. Although recordings were available, clinicians in the intervention arm missed some of the live case discussions, where the most practical guidance was shared, but not recorded. Preparing case presentations also required advanced effort, and time constraints may have led clinicians to underutilize this feature. These limitations could be mitigated in future implementations by protecting time for participation and simplifying case submission, which may enhance clinician engagement and patient care.

Finally, when our study was developed, few evidence-based treatment interventions for long COVID were available. Most published literature focused on symptom management rather than patient outcomes; thus we drew from the limited evidence to design a study centered on measuring outcomes in this population.

Despite these limitations, this study has several important strengths. To our knowledge, this is the first ECHO program evaluated for both effectiveness and implementation outcomes using randomization, patient-level data, and a comparison group. Unlike traditional educational approaches, LC&FIRP enabled real-time, case-based knowledge sharing to support timely, high-quality care. Additionally, the use of validated, multidimensional PROs, such as PROMIS-29, allowed for meaningful, patient-centered assessment and improved generalizability.

## 5 Conclusion

In conclusion, both study arms demonstrated improvements in clinical outcomes over the 12-month period, with the intervention arm achieving greater gains than the control arm in pain interference. Intervention patients also underwent fewer referrals and overall encounters than controls, indicating a potential health systems benefit to the intervention. Patients at FHCSD showed more improvement than those at UW, especially in domains such as dyspnea and social role functioning. These findings highlight the potential of structured, multidisciplinary educational programs that utilize an ECHO case-based virtual community of practice model, to enhance care delivery for complex, multi-systemic chronic illnesses like long COVID, ME/CFS, and other IACCs. While many questions remain about the mechanisms and optimal management of fatiguing illnesses, this study contributes evidence that clinician-focused case-based education, delivered through scalable, virtual community of practice platforms, can strengthen primary care capacity, especially in under-resourced primary care settings.

## Data Availability

The raw data supporting the conclusions of this article will be made available by the authors, without undue reservation.

## Supplementary material

The Supplementary Material for this article can be found online at: https://www.frontiersin.org/articles/10.3389/frhs.2026.1912240/full#supplementary-material

SUPPLEMENTARY TABLE 1 CONSORT Diagram.

SUPPLEMENTARY DATA SHEET 1 Supplementary Tables.
