# Supplementary material for: Effectiveness of an Extension for Community Health Outcomes case-based virtual community of practice model on patient outcomes for the management of long COVID and other fatiguing illnesses: a randomized control trial
Source: Front Health Serv. 2026 Oct 28;6:1912240. doi: 10.3389/frhs.2026.1912240 (PMC13752050; doi:10.3389/frhs.2026.1912240)
Supplement: Supplementary file 1 [file Datasheet1.docx]

**Supplementary Materials**

**Supplement 1: Individual PRO changes reaching MIC values**

Following the Pain Interference individual change score analysis reported in the main paper, we applied the same analysis on other PROMIS scales with near-significant (*P*<.10) average treatment effects in the LME analysis, considering both improvements and deterioration over 12 months. For each scale, we considered both the generally recommended MIC values (3 or 5) and the maximum MIC estimates from previous studies. Please note that this analysis only applies to the subset of FHCSD patients who completed both baseline and 12-month questionnaires.

Table S1. Number of patients (%) whose 12-month score changes exceed MIC values.

| **PROMIS Scale** | **Direction** | **Absolute**  **Change** | **Control**  n=174 | **Intervention**  n=145 | ***P*** |
| --- | --- | --- | --- | --- | --- |
| Anxiety | Improve | >= 3 | 73 (42%) | 70 (48%) | .31 |
|  |  | >= 3.5 | 72 (41%) | 70 (48%) | .26 |
|  | Deteriorate | >= 3 | 49 (28%) | 39 (27%) | .90 |
|  |  | >= 3.5 | 49 (28%) | 37 (26%) | .69 |
| Physical Function | Improve | >= 5 | 63 (36%) | 60 (41%) | .38 |
|  |  | >= 10 | 32 (18%) | 32 (22%) | .48 |
|  |  | >= 12.0 | 27 (16%) | 29 (20%) | .35 |
|  | Deteriorate | >= 5 | 22 (13%) | 20 (14%) | .87 |
|  |  | >= 10 | 7 (4%) | 5 (3%) | .99 |
|  |  | >= 12.0 | 7 (4%) | 1 (1%) | N/A |
| Pain Interference | Improve | >= 5 | 50 (29%) | 63 (43%) | .01 |
|  |  | >= 10 | 26 (15%) | 38 (26%) | .02 |
|  |  | >= 12.4 | 21 (12%) | 31 (21%) | .04 |
|  | Deteriorate | >= 5 | 33 (19%) | 24 (17%) | .63 |
|  |  | >= 10 | 8 (5%) | 9 (6%) | .72 |
|  |  | >= 12.4 | 8 (5%) | 7 (5%) | .99 |
| Sleep Disturbance | Improve | >= 2.4 | 72 (41%) | 73 (50%) | .14 |
|  |  | >= 3 | 69 (40%) | 71 (49%) | .12 |
|  | Deteriorate | >= 2.4 | 59 (34%) | 42 (29%) | .41 |
|  |  | >= 3 | 57 (33%) | 33 (23%) | .06 |
| Applied Cognition | Improve | >= 3 | 62 (36%) | 49 (34%) | .85 |
|  | Deteriorate | >= 3 | 29 (17%) | 29 (20%) | .49 |

**Supplement 2: Methods and results of exploratory moderation analysis**

Exploratory analysis of the moderation of intervention effects by the number of clinical encounters considered both the number of referrals received by a participant and the number of encounters the participant completed at FHCSD. Participants were first classified as either frequent or infrequent visitors based on the median number of received referrals or completed encounters, and this factor was then added to LME models to form a 3-way interaction with time and intervention/control assignment, as specified by the formula:

Score ~ Time + Arm + Freq + Time:Arm + Time:Freq + Arm:Freq + Time:Arm:Freq + ScoreBaseline + (1 | PatientID)

where Freq is the binary encounter frequency using either the number of referrals received or the number of encounters completed. Other terms are defined in the same way as models for intervention effect estimation.

Table S1 provides mean T-scores of FHCSD intervention and control patients on the PROMIS-29 Pain Interference scale at baseline and 12 months. Table S2 provides the results from interaction terms in the LME models described above, wherein most intervention effects are absorbed into the 3-way interaction term. Note that a higher score indicates more severe problems or concerns, therefore a negative estimate indicates a positive effect.

Table S2. Mean (SD) PROMIS-29 Pain Interference T-scores of FHCSD patients. BL: Baseline; 12M: 12 Month Follow-up

| **Arm, Time** | **Referrals Received** | | **Encounters Completed** | |
| --- | --- | --- | --- | --- |
|  | **< median** | **>= median** | **< median** | **>= median** |
| Control, BL | 58 (10) | 64 (9) | 58 (10) | 63.0 (9) |
| Control, 12M | 57 (12) | 62 (8) | 59 (10) | 61 (10) |
| Intervention, BL | 59 (10) | 63 (9) | 59 (10) | 63 (10) |
| Intervention, 12M | 54 (11) | 60 (10) | 54 (11) | 60 (10) |

Table S3. Interaction term results in LME models of PROMIS-29 Pain Interference scores.

| **Data** | **Interaction Term** | **Estimate** | ***P*** |
| --- | --- | --- | --- |
| Referrals received | Time:ArmINT  Time:FreqLow  ArmINT:FreqLow  Time:ArmINT:FreqLow | 0.18  0.40  0.62  -4.87 | .89  .77  .59  .011 |
| Encounters completed | Time:ArmINT  Time:FreqLow  ArmINT:FreqLow  Time:ArmINT:FreqLow | -0.58  1.35  -0.37  -3.87 | .63  .33  .75  .049 |

**Supplement 3: CDC symptom screener results**

Calculation of symptom severity scores uses the frequency score $\times$ intensity score algorithm of the CDC symptom screener(27). Severity scores range from 0 to 16, where 0 represents absence or resolution of a symptom. A higher score indicates that the symptom is more frequent or intense.

Table S4. Symptom prevalence and severity scores at baseline.

| **Symptom** | **All**  N=586 | **Control**  n=298 | **Intervention**  n=288 | ***P*** |
| --- | --- | --- | --- | --- |
| Fatigue, tiredness, or exhaustion | | | | |
| Yes, n (%)  Severity score, mean (SD) | 556 (94.9)  10.6 (4.8) | 282 (94.6)  10.9 (4.8) | 274 (95.1)  10.4 (4.8) | .78  .22 |
| Muscle pain, muscle cramps, or muscle aches | | | | |
| Yes, n (%)  Severity score, mean (SD) | 429 (73.2)  6.9 (5.7) | 218 (73.2)  6.9 (5.7) | 211 (73.3)  6.8 (5.7) | .98  .97 |
| Joint pain | | | | |
| Yes, n (%)  Severity score, mean (SD) | 398 (67.9)  6.4 (5.7) | 206 (69.1)  6.4 (5.5) | 192 (66.7)  6.3 (5.9) | .52  .91 |
| Unrefreshing sleep | | | | |
| Yes, n (%)  Severity score, mean (SD) | 420 (71.7)  7.1 (5.9) | 213 (71.5)  7.2 (6.0) | 207 (71.9)  7.0 (5.8) | .92  .74 |
| Problems getting to sleep, sleeping through the night, or waking up on time | | | | |
| Yes, n (%)  Severity score, mean (SD) | 446 (76.1)  7.7 (5.8) | 221 (74.2)  7.6 (5.9) | 225 (78.1)  7.8 (5.7) | .26  .64 |
| Forgetfulness or memory problems | | | | |
| Yes, n (%)  Severity score, mean (SD) | 437 (74.6)  7.0 (5.9) | 221 (74.2)  7.0 (5.8) | 216 (75.0)  7.1 (5.9) | .82  .77 |
| Difficulty thinking or concentrating | | | | |
| Yes, n (%)  Severity score, mean (SD) | 457 (78.0)  7.3 (5.7) | 230 (77.2)  7.0 (5.7) | 227 (78.8)  7.5 (5.8) | .63  .29 |
| Dizziness or lightheadedness | | | | |
| Yes, n (%)  Severity score, mean (SD) | 436 (74.4)  4.9 (4.7) | 216 (72.5)  4.4 (4.5) | 220 (76.4)  5.4 (4.9) | .28  .02 |
| Post-exertional malaise^a^ | | | | |
| Yes, n (%) | 412 (70.3) | 201 (67.4) | 211 (73.3) | .12 |

^a^Post-exertional malaise was measured by a yes, no, or do not know question asking whether any of the symptoms get worse for at least 24 hours after activity.

Symptom follow-ups were conditional. Patients were asked about a specific symptom only if they had reported that symptom at baseline. Accordingly, the 12-month symptom analysis included only patients who answered Yes for a specific symptom at baseline and responded to the symptom survey at 12 months. Thus, the 12-month results describe symptom persistence among a subset of baseline symptom reporters, and are not directly comparable to baseline results. Because the number of patients reporting each symptom at baseline differed, the eligible sample size also differed across symptoms at 12 months.

Table S5. Symptom persistence and severity scores at 12 months.

| **Symptom** | **All** | **Control** | **Intervention** | ***P*** |
| --- | --- | --- | --- | --- |
| Fatigue, tiredness, or exhaustion | | | | |
| Sample size  Persistent, n (%)  Severity score, mean (SD) | 232  178 (76.7)  7.3 (5.4) | 127  99 (78.0)  7.8 (5.3) | 105  79 (75.2)  6.8 (5.5) | .63  .15 |
| Muscle pain, muscle cramps, or muscle aches | | | | |
| Sample size  Persistent, n (%)  Severity score, mean (SD) | 183  139 (76.0)  6.9 (5.3) | 103  79 (76.7)  7.8 (5.5) | 80  60 (75.0)  5.8 (4.9) | .79  .01 |
| Joint pain | | | | |
| Sample size  Persistent, n (%)  Severity score, mean (SD) | 172  127 (73.8)  7.0 (5.2) | 98  72 (73.5)  7.6 (5.4) | 74  55 (74.3)  6.1 (4.8) | .90  .06 |
| Unrefreshing sleep | | | | |
| Sample size  Persistent, n (%)  Severity score, mean (SD) | 177  98 (55.4)  5.9 (5.6) | 105  62 (59.0)  6.2 (5.4) | 72  36 (50.0)  5.3 (5.9) | .23  .33 |
| Problems getting to sleep, sleeping through the night, or waking up on time | | | | |
| Sample size  Persistent, n (%)  Severity score, mean (SD) | 197  128 (65.0)  6.4 (5.5) | 110  74 (67.3)  6.6 (5.4) | 87  54 (62.1)  6.2 (5.7) | .45  .61 |
| Forgetfulness or memory problems | | | | |
| Sample size  Persistent, n (%)  Severity score, mean (SD) | 187  128 (68.4)  6.0 (4.9) | 109  73 (67.0)  6.1 (5.1) | 78  55 (70.5)  5.8 (4.6) | .61  .64 |
| Difficulty thinking or concentrating | | | | |
| Sample size  Persistent, n (%)  Severity score, mean (SD) | 194  133 (68.6)  5.8 (4.8) | 109  79 (72.5)  6.0 (4.8) | 85  54 (63.5)  5.5 (4.8) | .18  .50 |
| Dizziness or lightheadedness | | | | |
| Sample size  Persistent, n (%)  Severity score, mean (SD) | 179  105 (58.7)  4.1 (4.5) | 96  60 (62.5)  4.6 (4.4) | 83  45 (54.2)  3.5 (4.5) | .28  .26  .13 |
| Post-exertional malaise^a^ | | | | |
| Sample size  Persistent, n (%) | 175  126 (72.0) | 97  73 (75.3) | 78  53 (67.9) | .28 |

^a^Post-exertional malaise was measured by a yes, no, or do not know question asking whether any of the symptoms get worse for at least 24 hours after activity.

**Supplement 4: Cohort comparison results between FHCSD and UW**

Please note that this analysis only includes patients who completed both baseline and 12-month questionnaires at FHCSD and UW due to limited data availability in the UW cohort. “Match” in the following tables refers to propensity score matching.

Table S6. FHCSD and UW patient characteristics.

| **Characteristics** | **FHCSD**  n=319 | **UW**  n=238 | ***P*** | | **Effect Size After Match** |
| --- | --- | --- | --- | --- | --- |
|  |  |  | **Before Match** | **After Match** |  |
| Age, mean (SD) | 48.3 (12.7) | 48.0 (13.2) | .82 | .87 | 0.01 |
| Sex, n (%)  Female  Male | 233 (73.5%)  84 (26.5%) | 162 (68.1%)  75 (31.5%) | .40 | .99 | 0+ |
| Hospitalized due to COVID-19  Yes, n (%) | 73 (22.9%) | 21 (8.8%) | <.001 | .99 | 0+ |
| Admitted to ICU due to COVID-19  Yes, n (%) | 39 (12.2%) | 7 (2.9%) | <.001 | .10 | 0.09 |
| Intubated due to COVID-19  Yes, n (%) | 19 (6.0%) | 6 (2.5%) | .08 | .53 | 0.03 |

Table S7. Patient-reported outcomes^a^ of FHCSD and UW patients at baseline and 12 months. No propensity score matching. C: control. I: intervention.

|  | **Baseline, mean (SD)** | **C**  n=174 | **I**  n=145 | **UW**  n=238 | ***P*** | | |
| --- | --- | --- | --- | --- | --- | --- | --- |
|  |  |  |  |  | **C vs I** | **C vs UW** | **I vs UW** |
|  | PROMIS-29 Depression | 54 (11) | 54 (10) | 56 (9) | .66 | .02 | .06 |
|  | PROMIS-29 Anxiety | 56 (11) | 58 (11) | 59 (10) | .20 | .009 | .29 |
|  | PROMIS-29 Fatigue | 62 (10) | 63 (9) | 67 (9) | .32 | <.001 | <.001 |
|  | PROMIS-29 Physical Function | 37 (8) | 37 (7) | 40 (8) | .84 | <.001 | <.001 |
|  | PROMIS-29 Pain Interference | 62 (10) | 62 (9) | 59 (10) | .68 | .002 | <.001 |
|  | PROMIS-29 Social Roles | 44 (11) | 45 (11) | 39 (9) | .56 | <.001 | <.001 |
|  | PROMIS-29 Sleep Disturbance | 58 (8) | 58 (8) | 56 (8) | .55 | .14 | .04 |
|  | PROMIS Dyspnea | 58 (11) | 58 (11) | 54 (10) | .59 | <.001 | .001 |
|  | PROMIS Applied Cognition | 43 (11) | 43 (11) | 35 (10) | .72 | <.001 | <.001 |
|  | GAD-7 (Anxiety) | 6.7 (5.8) | 7.4 (5.3) | N/A | .26 | N/A | N/A |
| **12M, mean (SD)** | | **C**  n=174 | **I**  n=145 | **UW**  n=238 | ***P*** | | |
|  |  |  |  |  | **C vs I** | **C vs UW** | **I vs UW** |
| PROMIS-29 Depression | | 52 (11) | 52 (11) | 54 (10) | .88 | .07 | .047 |
| PROMIS-29 Anxiety | | 55 (12) | 54 (12) | 56 (10) | .58 | .21 | .047 |
| PROMIS-29 Fatigue | | 57 (12) | 57 (12) | 63 (11) | .58 | <.001 | <.001 |
| PROMIS-29 Physical Function | | 40 (10) | 41 (10) | 42 (9) | .33 | .07 | .49 |
| PROMIS-29 Pain Interference | | 60 (10) | 58 (11) | 56 (11) | .02 | <.001 | .23 |
| PROMIS-29 Social Roles | | 51 (12) | 51 (12) | 42 (10) | .82 | <.001 | <.001 |
| PROMIS-29 Sleep Disturbance | | 57 (9) | 56 (9) | 55 (8) | .21 | .04 | .54 |
| PROMIS Dyspnea | | 51 (12) | 49 (12) | 51 (12) | .15 | .72 | .18 |
| PROMIS Applied Cognition | | 49 (12) | 47 (13) | 38 (12) | .23 | <.001 | <.001 |
| GAD-7 (Anxiety) | | 5.8 (5.3) | 5.7 (5.8) | 5.7 (5.7) | .91 | .90 | .99 |

^a^On GAD-7 and all PROMIS subscales except Physical Function, Social Roles, and Applied Cognition, a higher score indicates more severe problems or concerns. On PROMIS Physical Function, Social Roles, and Applied Cognition scales, a lower score indicates more severe problems or concerns.

Table S8. Patient-reported outcomes^a^ of FHCSD (control and intervention combined) and UW patients at baseline and 12 months.

| **Baseline, mean (SD)** | **FHCSD**  n=319 | **UW**  n=238 | ***P*** | | **Effect Size After Match** |
| --- | --- | --- | --- | --- | --- |
|  |  |  | **Before Match** | **After Match** |  |
| PROMIS-29 Depression | 54 (11) | 56 (9) | .008 | <.001 | 0.29 |
| PROMIS-29 Anxiety | 57 (11) | 59 (10) | .023 | <.001 | 0.24 |
| PROMIS-29 Fatigue | 62 (9) | 67 (9) | <.001 | <.001 | 0.54 |
| PROMIS-29 Physical Function | 37 (8) | 40 (8) | <.001 | <.001 | 0.31 |
| PROMIS-29 Pain Interference | 62 (9) | 59 (10) | <.001 | .002 | -0.27 |
| PROMIS-29 Social Roles | 45 (11) | 39 (9) | <.001 | <.001 | -0.61 |
| PROMIS-29 Sleep Disturbance | 58 (8) | 56 (8) | .04 | .002 | -0.26 |
| PROMIS Dyspnea | 58 (11) | 54 (10) | <.001 | <.001 | -0.31 |
| PROMIS Applied Cognition | 43 (11) | 35 (10) | <.001 | <.001 | -0.74 |
| GAD-7 (Anxiety) | 7.0 (5.6) | N/A | N/A | N/A | N/A |
| **12M, mean (SD)** | **FHCSD**  n=319 | **UW**  n=238 | ***P*** | | **Effect Size After Match** |
|  |  |  | **Before Match** | **After Match** |  |
| PROMIS-29 Depression | 52 (11) | 54 (10) | .019 | <.001 | 0.29 |
| PROMIS-29 Anxiety | 54 (12) | 56 (10) | .055 | .034 | 0.18 |
| PROMIS-29 Fatigue | 57 (12) | 63 (11) | <.001 | <.001 | 0.53 |
| PROMIS-29 Physical Function | 41 (10) | 42 (9) | .15 | .68 | 0.03 |
| PROMIS-29 Pain Interference | 59 (10) | 56 (11) | .003 | .004 | -0.25 |
| PROMIS-29 Social Roles | 51 (12) | 42 (10) | <.001 | <.001 | -0.95 |
| PROMIS-29 Sleep Disturbance | 56 (9) | 55 (8) | .11 | .023 | -0.20 |
| PROMIS Dyspnea | 50 (12) | 51 (12) | .52 | .85 | -0.02 |
| PROMIS Applied Cognition | 48 (12) | 38 (12) | <.001 | <.001 | -0.79 |
| GAD-7 (Anxiety) | 5.8 (5.5) | 5.7 (5.7) | .98 | .91 | 0.01 |

^a^On GAD-7 and all PROMIS subscales except Physical Function, Social Roles, and Applied Cognition, a higher score indicates more severe problems or concerns. On PROMIS Physical Function, Social Roles, and Applied Cognition scales, a lower score indicates more severe problems or concerns.

Table S9. Estimated cohort differences^a^ in patient-reported outcome^b^ changes over 12 months from weighted LME models.

| **Scale** | **Estimate** | ***P*** |
| --- | --- | --- |
| PROMIS-29 Depression | 0.21 | .80 |
| PROMIS-29 Anxiety | -0.53 | .55 |
| PROMIS-29 Fatigue | 1.03 | .24 |
| PROMIS-29 Physical Function | -2.31 | .002 |
| PROMIS-29 Pain Interference | 0.09 | .92 |
| PROMIS-29 Social Roles | -3.93 | <.001 |
| PROMIS-29 Sleep Disturbance | 0.47 | .50 |
| PROMIS Dyspnea | 3.72 | <.001 |
| PROMIS Applied Cognition | -0.63 | .48 |

^a^Cohort difference estimates come from the interaction term between time and UW. These estimates reflect the UW cohort’s average relative changes in the outcomes compared to the FHCSD cohort.

^b^On all PROMIS subscales except Physical Function, Social Roles, and Applied Cognition, a higher score indicates more severe problems or concerns. Therefore, a negative estimate on these scales indicates more improvements or less deterioration in the UW cohort than the FHCSD cohort. On PROMIS Physical Function, Social Roles, and Applied Cognition scales, a lower score indicates more severe problems or concerns. Therefore, a negative estimate on these scales indicates less improvements or more deterioration in the UW cohort than the FHCSD cohort.
